# Chromium Environment within Cr-Doped Silico-Aluminophosphate Molecular Sieves from Spin Density Studies

**DOI:** 10.1021/acs.jpcc.0c09484

**Published:** 2021-04-07

**Authors:** Yu-Kai Liao, Paolo Cleto Bruzzese, Martin Hartmann, Andreas Pöppl, Mario Chiesa

**Affiliations:** † Dipartimento di Chimica, Università di Torino and NIS Centre, Via Pietro Giuria 7, 10125 Torino, Italy; ‡ Felix Bloch Institute for Solid State Physics, Universität Leipzig, Linnéstr. 5, 04103 Leipzig, Germany; § Erlangen Center for Interface Research and Catalysis (ECRC), Egerlandstr. 3, 91058 Erlangen, Germany

## Abstract

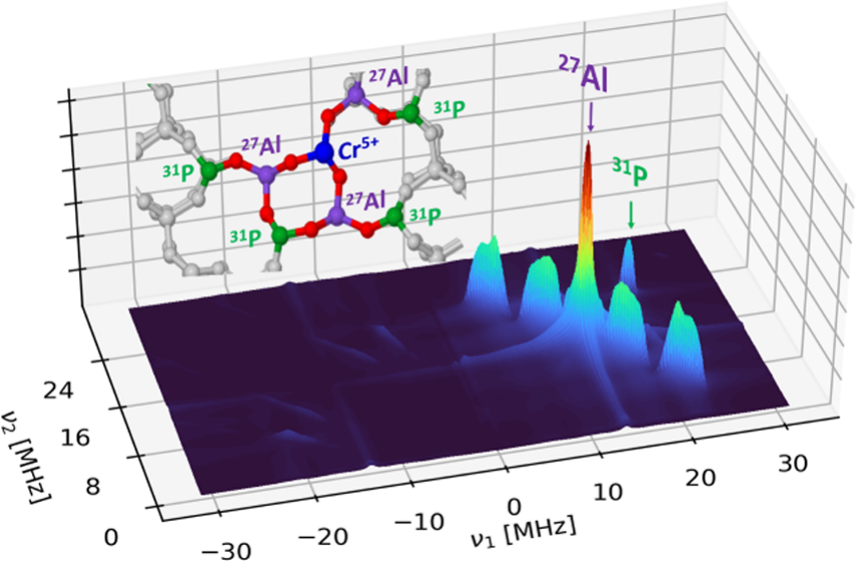

X-/Q-band electron paramagnetic resonance (EPR) and hyperfine sublevel correlation (HYSCORE) spectroscopies have been employed, in conjunction with density functional theory (DFT) modeling, to determine the location of Cr^5+^ions in SAPO-5 zeotype materials. The interaction of the unpaired electron of the paramagnetic Cr^5+^ species with ^27^Al could be resolved, allowing for the first detailed structural analysis of Cr^5+^ paramagnetic ions in SAPO materials. The interpretation of the experimental results is corroborated by DFT modeling, which affords a microscopic description of the system investigated. The EPR-active species is found to be consistent with isolated Cr^5+^ species isomorphously substituted in the framework at P^5+^ sites.

## Introduction

**Figure 1:**
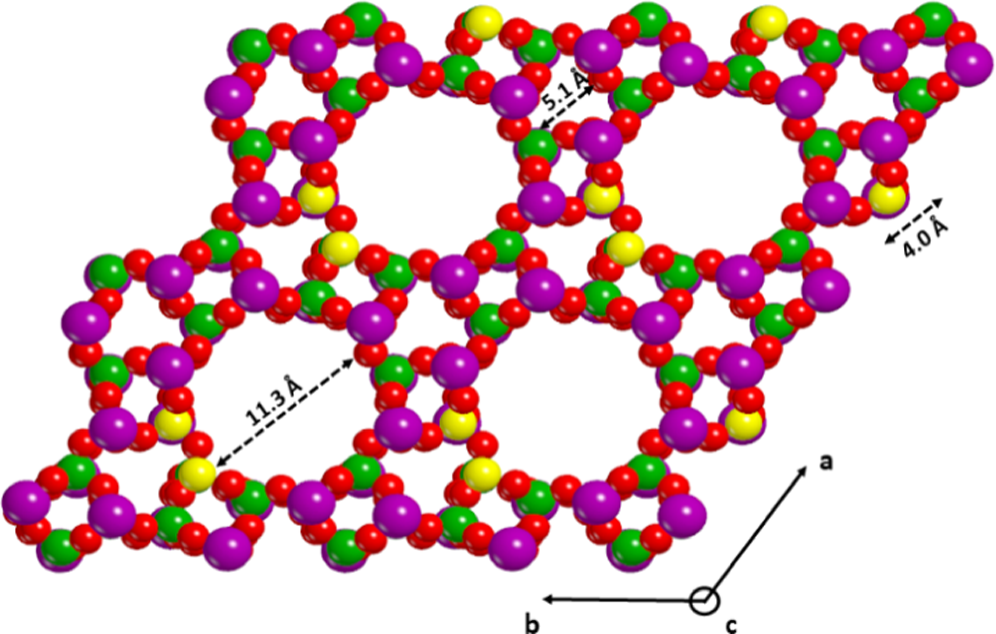
View along the *c*-axis of the SAPO-5 space-filling periodic model. The dashed arrows report the size of the different pores. Si, Al, P, and O are in yellow, violet, green, and red, respectively.

Aluminophosphate molecular sieves (AlPOs)^1^—characterized by neutral lattices of alternating TO_4_ (T = Al or P) tetrahedra—form a class of microporous crystalline materials comparable to zeolites, featuring characteristic properties linked to their unique composition. One of the peculiar features of AlPOs is the electroneutrality of the framework, which limits their use as acid catalysts. However, Brønsted acidity can be introduced by the incorporation of heteroelements such as silicon, leading to so-called SAPOs. The SAPO maintains the same overall structure as the parent AlPO, although local structural changes are found as a consequence of the substitution.^2^ The SAPO-5 structure with the relative pore dimensions is shown in Figure 1.

In the same way, redox functionalities can be tuned through the insertion of specific transition-metal ions (TMI), and a range of TMI-substituted SAPOs have been synthesized, displaying characteristic properties.^3^ The combination of these two strategies, leading to the simultaneous presence of Brønsted and redox sites, is a viable path to synthesize selective catalysts with a peculiar bifunctional character, where the reactivity of redox and acidic functionalities is combined with the high surface area and the unique spatial constraints imposed by the molecular dimensions of the porous network.^4^

Among the large variety of different TMIs, Cr-doped SAPOs are of interest due to the specific activity of isolated Cr sites toward different catalytic processes.^5^ In general, isomorphous substitution of heterometals into molecular sieves largely depends on the nature of the metal, and chromium belongs to the group for which substitution is difficult and evidence for isomorphous substitution is often indirect.^6^ Substitution of Cr into AlPO4-5 was claimed for the first time by Flanigen et al.^7^ Later, Weckhuysen and Schoonheydt challenged the framework incorporation of Cr^3+^ mainly because of its preference for octahedral coordination.^8,9^ Through specific synthetic protocols, the framework incorporation of Cr^3+^ has been reported,^6^ and the unusual tetrahedral coordination of Cr^3+^ is confirmed by detailed X-ray absorption spectroscopic studies.^10^ In the same systems, a certain amount of Cr^5+^ was detected with UV–vis and/or electron paramagnetic resonance (EPR) spectroscopy but the question of whether this was a framework species substituted for P or a type of surface species anchored to the framework remains open.^11^ In the case of CrAPSO-5, Zhu and Kevan, based on electron spin echo envelope modulation (ESEEM) studies,^12^ proposed that after calcination, Cr^5+^ species substitute for P^5+^ framework ions. In CrSAPO systems, the specific site (Al or P) at which Cr substitutes largely depends on its oxidation state. Moreover, the overall picture is complicated by the different possible substituting sites for Si, for which the tendency to form isolated sites or islands comprising Si–O–Si bridges has been established.^2,13^ In the case of Cr, both Cr^3+^ and Cr^5+^ can potentially be present,^12^ both featuring a paramagnetic electronic structure, with *S* = 3/2 and *S* = 1/2 spin states for Cr^3+^and Cr^5+^, respectively. Unusual tetrahedral coordination of Cr^3+^ in microporous aluminophosphates has been demonstrated,^10^ while less attention has been given to higher oxidation states. The goal of this work is therefore the detailed spectroscopic characterization of the coordination environment of Cr^5+^ in CrSAPO-5.

One of the most potent descriptors of the local environment of paramagnetic transition-metal ions is EPR and the associated hyperfine techniques of electron nuclear double resonance (ENDOR) and hyperfine sublevel correlation (HYSCORE) spectroscopies, which can provide sub-MHz resolution, allowing the coupling of the sensitivity and selectivity of EPR with the resolution of nuclear magnetic resonance (NMR). In particular, for TMIs incorporated in various aluminophosphate molecular sieves, analysis of the hyperfine interactions due to nearby ^27^Al (*I* = 5/2),^14−16^^29^Si (*I* = 1/2),^17^ and ^31^P (*I* = 1/2)^18,19^ nuclei from the framework allows obtaining direct information about the metal ion location. Zhu and Kevan reported EPR and electron spin echo modulation (ESEEM) studies on CrSAPO-5, proposing that small amounts of Cr (0.043 mol %) substitute for P^5+^, as Cr^3+^ in the as-prepared crystals and as Cr^5+^ after calcination.^12^ The framework incorporation of Cr^5+^ was derived from weak ^31^P ESEEM signals originating from the fourth coordination sphere. However, the magnetic interactions of the transition-metal ions with the ^27^Al nuclei in the second coordination sphere were missing, and consequently, no detailed structural models for the Cr^5+^ incorporation site could be given here. Moreover, Kornatowski et al. stressed the importance of the synthesis conditions in driving the framework incorporation of Cr in specific oxidation states,^6^ pointing to the importance of reliable analytical techniques capable of giving detailed information in this regard.

In this work, we employ EPR and HYSCORE spectroscopies in conjunction with density functional approximation (DFT) calculations to monitor the incorporation of small amounts of Cr^5+^ ions (<0.043 mol %) in the SAPO-5 system, featuring an AFI framework type composed of 12-membered rings aligned in parallel. In particular, we report for the first time the observation of large ^27^Al hyperfine couplings, which, combined with the observation of remote ^31^P couplings in the HYSCORE experiments, provide direct evidence for framework substitution of Cr at P sites. For the analysis of the ^27^Al hyperfine couplings, periodic and cluster DFT computations were employed to interpret the experimentally obtained data in terms of microscopic model structures for Cr^5+^incorporation in SAPO-5 materials, also considering different possible distributions of Si ions in the framework.

## Materials and Methods

### Sample Preparation

**Figure 2:**
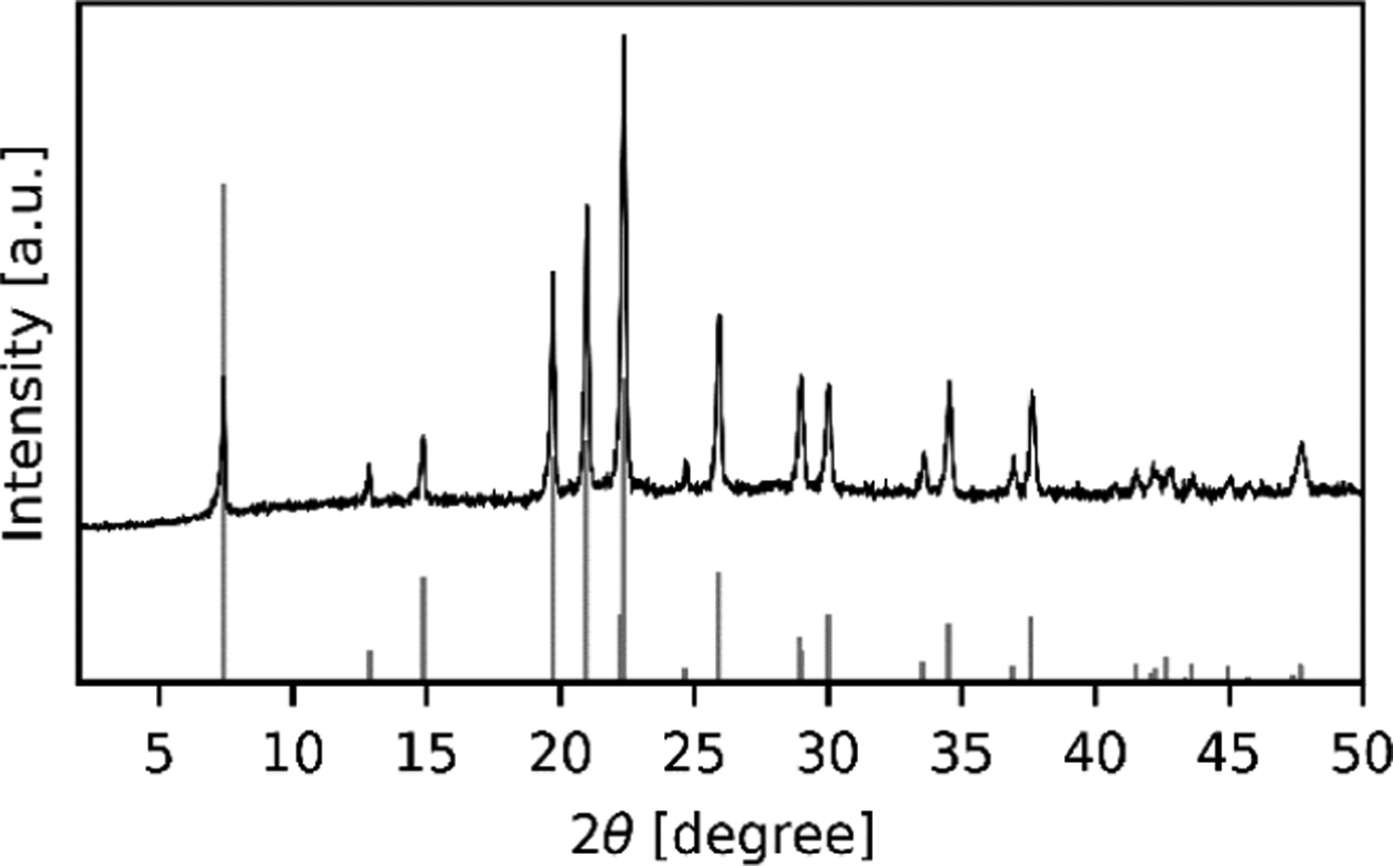
PXRD diffraction pattern of CrSAPO-5 after the removal of the template. Gray bars indicate the theoretical reflections of the AFI structure from the Database of Zeolite Structures.^22^

The CrSAPO-5 was prepared by hydrothermal synthesis according to the method reported by Zhu and Kevan.^12^ The sample was synthesized with 0.043 mol % chromium. The synthesis started by mixing 20.400 g of aluminum isopropoxide and 20.000 g of distilled water and stirring until the slurry was homogeneous. Then, a mixture of 0.027 g of CrCl_3_·6H_2_O dissolved in 4.185 g of distilled water and 12.106 g of 85% phosphoric acid was added dropwise to the slurry. After stirring for 1 h, a mixture of 0.900 g of fumed silica and 10.000 g of distilled water was added dropwise and stirred for another 0.5 h. Afterward, 7.150 g of tripropylamine was added dropwise to the mixture and stirred overnight to ensure the homogeneity. The mixture was transferred to a 100 mL Teflon-lined autoclave and heated at 220 °C for 48 h. The autoclave was quenched after synthesis, and the solid product was recovered by centrifugation, repeatedly washed with water, and dried at 80 °C overnight. To remove the template, the sample was calcined at 550 °C in nitrogen flow for 12 h and in air flow for 6 h. The AFI structure of the final product was verified by the powder X-ray diffraction pattern (Figure 2) obtained on an X’Pert Pro diffractometer (equipped with an X’Celerator detector by PANalytical using Cu Kα radiation) with the same reflection as shown in the previous studies.^20,21^ Elemental analysis of the CrSAPO-5 material was carried out by inductively coupled plasma atomic emission spectroscopy (ICP-AES) measurements, leading to the following elemental composition: 18.8 wt % Al, 18.6 wt % P, 1.1 wt % Si, and 0.035 wt % Cr. The ICP-OES was carried out with a Ciros-CCD by Spectro. The sample was digested in a mixture of 4 mL of HCl (37%), 2 mL of HNO_3_ (65%), and 8 mL of HF (40%) using a microwave oven for heating to 200 °C. After calcination, the sample was dehydrated at 120 °C under dynamic vacuum until the pressure was stabilized below the detectable limit.

### Electron Paramagnetic Resonance Spectroscopy

Continuous wave EPR (CW-EPR) experiments at the X-band (∼9.5 GHz) were performed on a Bruker EMX EPR spectrometer equipped with a super-high-Q cavity. The EPR cell was placed in a finger Dewar filled with liquid nitrogen to perform the measurement at 77 K.

Pulsed EPR experiments at the Q-band (∼33.8 GHz) were performed on a Bruker ELEXYS 580 EPR spectrometer with a liquid-helium cryostat from Oxford Inc. and an ER035M NMR gaussmeter from Bruker.

Electron spin echo-detected (ESE-detected) field sweep experiments were performed at *T* = 50 K with the pulse sequence π/2-τ-π-τ-*echo*. The pulse lengths were 16 ns for *t*_π/2_ and 32 ns for *t*_π_, while *t*_τ_ was 200 ns and repetition rate was 1 kHz.

The four-pulse HYSCORE^23^ experiments were performed at 1222.5 mT and *T* = 40 K with the pulse sequence π/2-τ-π/2-*t*_1_-π-*t*_2_-π/2-τ-echo. The pulse lengths were 14 ns for *t*_π/2_ and 28 ns for *t*_π_. The increment of the time intervals *t*_1_ and *t*_2_ was 16 ns from 100 to 3300 ns, and the repetition rate was 1.33 kHz. Three different interpulse delays τ (110, 116, and 146 ns) were used to avoid blind spots in the spectra.

The six-pulse HYSCORE^24,25^ experiments were performed at 1224.1 mT and *T* = 40 K with the pulse sequence (π/2)_*x*_-τ_1_-(π)_*x*_-τ_1_-(π/2)_*y*_-*t*_1_-(π)_*y*_-*t*_2_-(π/2)_*y*_-τ_2_-(π)_*y*_-τ_2_-echo. The pulse lengths were 14 ns for *t*_π/2_ and 28 ns for *t*_π_. The increment of the time intervals *t*_1_ and *t*_2_ was 16 ns from 100 to 3300 ns, and the repetition rate was 980 Hz. With τ_1_ = τ_2_ = τ, two different interpulse delays τ (110 and 146 ns) were used to avoid blind spots in the spectra. An eight-step phase cycle was adopted to eliminate unwanted echoes.

EPR and HYSCORE spectra were simulated using the EasySpin toolbox.^26^

### Models and Computational Details

#### Periodic and Cluster Models of the CrSAPO-5 Catalyst

The CrSAPO-5 structure was simulated using a periodic approach that provides more reliable models than the molecular cluster ones because of the better description of the crystalline environment of the silico-aluminophosphate material. Starting from the AlPO-5 framework (AFI),^1^ we considered three periodic models with different Si distributions. For each model, we performed a full geometry optimization in which both internal coordinates and lattice vectors had been relaxed in the P1 space group, without any symmetry constraint.

The periodic DFT study has been complemented with molecular cluster calculations to estimate the ***g***-tensor of Cr^5+^ and the relative orientations of the ^27^Al hyperfine interactions (hfi) with respect to the ***g*** frame. Three cluster models including Cr together with the surrounding atoms up to the fourth coordination shell had been removed from the corresponding optimized periodic structures. The involvement of the fourth sphere around chromium was necessary to obtain a good representability of the cluster with respect to the periodic models as proved by the similarity of the Cr spin density values. The dangling bonds were saturated with hydrogen atoms oriented along the broken bonds to maintain the local environment as in the optimized periodic models. Thus, no further geometry optimization of the cluster models was performed: the ***g***-tensor was computed maintaining the same atomic coordinates as the ones in the relaxed periodic structures. The net charge on the clusters was set to 0 in a doublet spin state.

#### Computational Details

Periodic calculations were performed using the distributed parallel version of CRYSTAL17 code (PCRYSTAL)^27^ within the density functional theory (DFT) approximation adopting the hybrid B3LYP method, Becke’s three-parameter exchange functional and the correlation functional from Lee, Yang, and Parr.^28,29^ The semiempirical dispersion corrections for the vdW interactions were treated by employing the Grimme approach in the so-called DFT-D3 method^30^ including also a three-body correction,^31^ as implemented in the CRYSTAL17 software package. The new version of the pob-TZVP basis set, denoted as pob-TZVP-rev2,^32^ was used for all of the elements during the geometry relaxation of both atomic coordinates and cell vectors. For the magnetic property prediction, a single point calculation with the same level of theory was carried out except for Al atoms, for which the aug-cc-pVTZ-J basis set^33^ was used. This one is characterized by a particularly rich and flexible core region, and, thus, it can better describe the electron density at the nuclei, fundamental for the accurate computation of the isotropic component of the hyperfine coupling tensor. Furthermore, for Al atoms, the primitive Gaussians with exponents lower than 0.06 were removed to avoid linear dependency in the self-consistent cycle (SCF).

A default pruned grid built according to the Gauss–Legendre quadrature and Lebedev schemes having 75 radial points and a maximum number of 974 angular points in regions relevant for chemical bonding has been used. The tolerances that control the accuracy of the calculation of the bioelectronic Coulomb and exchange series are selected according to the entity of overlap between two atomic orbitals (AO). In this work, all of the truncation criteria were set up to the value of 8 (ITOL1, ITOL2, ITOL3, and ITOL4) except for the criterion of pseudo-overlap of the HF exchange series (ITOL5), which was fixed to 30 (CRYSTAL17 Manual). A shrink factor equal to 4 was used to diagonalize the Hamiltonian matrix in at least 36 *k*-points of the first Brillouin zone. The default value of mixing (30%) of the Kohn–Sham (KS) matrix at a cycle with the previous one was adopted. The threshold in energy variation of self-consistent field (SCF) cycles was set to 10^–8^ Hartree for both geometry optimization and magnetic property evaluation. The spin of the periodic models, defined as the difference of the number of α and β electrons, was not locked to 1 to leave the SCF procedure to converge to its natural solution, which was a doublet spin state of the system wavefunction.

Molecular cluster calculations were carried out using the ORCA (v4.2.1) code,^34^ which is equipped with a specific EPR/NMR module that allows the ab initio assessment of the ***g***-tensor. The same exchange–correlation functional and corrections for dispersion were used (B3LYP-D3) with the inclusion of the three-body correction. However, specifically developed for magnetic properties, Gaussian basis functions were employed for the elements of the cluster models. The IGLO-III^35^ basis set was adopted for P, H, O, and Si atoms, the recommended CP(PPP)^36^ for Cr atoms, and the complete version of the aug-cc-pVTZ-J^33^ for Al atoms. The SCF convergence criteria were increased up to 10^–8^ Hartree. An integration grid composed of 770 radial points in agreement with the Lebedev scheme was chosen for all of the atoms.

## Results and Discussion

### EPR Characterization

**Figure 3:**
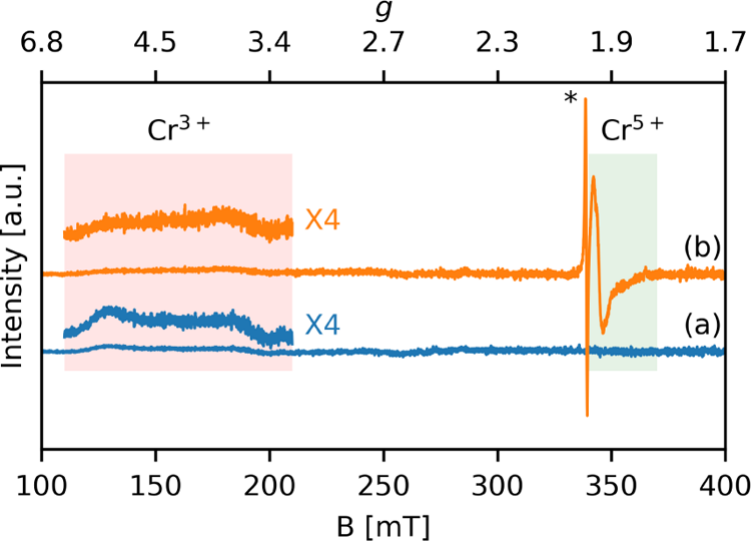
X-band CW-EPR spectra of (a) as-synthesized and (b) calcined CrSAPO-5 samples. Spectra were recorded at *T* = 77 K. The red rectangle shows the region of Cr^3+^ signals, and the green rectangle shows the region of the Cr^5+^ signal. The radical impurity signal of the calcined CrSAPO-5 is marked with an asterisk.

The CW-EPR spectrum of the as-synthesized CrSAPO-5 measured at the X-band (Figure 3a) showed a broad absorption between 120 and 200 mT (*g*_eff_ = 5.2–3.2), characteristic of Cr^3+^ species in CrAPO-5^11^ and CrAPSO-11^37^ and associated with octahedrally distorted coordinations.^8,12,37,38^

After calcination of the sample (Figure 3b), part of the Cr^3+^ signal can still be observed, indicating the presence of highly stable Cr^3+^, which can be associated with the isomorphous substitution, in agreement with previous reports.^11^ Moreover, the spectrum shows a new pseudoaxial signal centered at about *g* = 1.97, along with a sharp signal at *g* = 2.003 due to a radical species formed during removal of the template by calcination.^11^ The axial signal at *g* = 1.97 is assigned to a Cr^5+^ species forming a CrO_4_^3–^ unit (*S* = 1/2). The computer simulation of the spectrum reported in Figure S1a indicates a rhombic ***g***-tensor with *g*_*z*_ = 1.985 ± 0.001, *g*_*y*_ = 1.972 ± 0.001, and *g*_*x*_ = 1.953 ± 0.003, in agreement with the results of DFT modeling (vide infra). The same set of spin Hamiltonian parameters was used to fit the Q-band electron spin echo (ESE)-detected EPR spectrum in Figure S1b.

**Figure 4:**
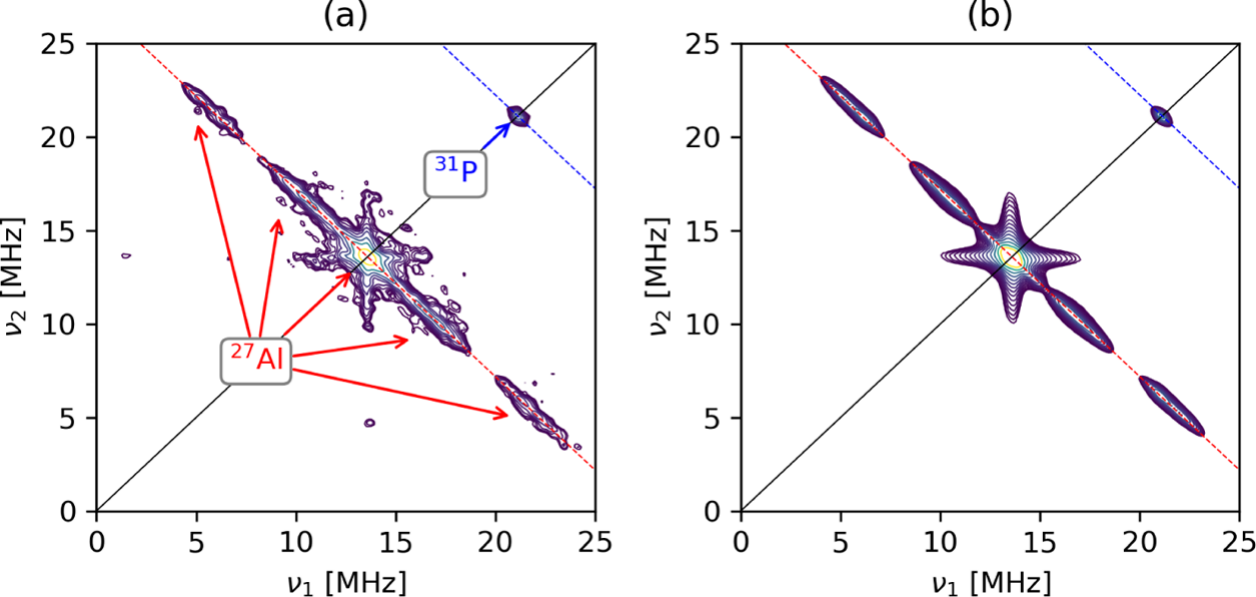
(a) Q-band ^27^Al six-pulse HYSCORE spectrum of CrSAPO-5 recorded at a magnetic field setting corresponding to the maximum echo intensity (arrow in Figure S1b) and *T* = 40 K. The spectrum is the sum of individual spectra recorded with different τ values (τ = 110 and 146 ns) and summed after the Fourier transform. The red arrows indicate ^27^Al cross-peaks. The red and blue dashed lines indicate the Larmor frequencies of ^27^Al (ν = 13.59 MHz) and ^31^P (ν = 21.12 MHz). (b) Simulation of the experimental six-pulse HYSCORE spectrum using the three groups of ^27^Al hfi tensors and the estimated ^31^P hfi tensor listed in Table 1.

To establish the CrO_4_^3–^ environment, Q-band six-pulse HYSCORE experiments were performed at a field position ***B***_0_ = 1224.1 mT corresponding to the maximum intensity of the ESE-detected EPR spectrum (Figure S1b). Six-pulse HYSCORE spectra give substantially enhanced peak intensities for weakly modulating nuclei as in the case of the Q-band measurement of hyperfine interactions characterized by a small anisotropic component. This sequence was therefore adopted as an alternative to standard HYSCORE experiments.^25^ (Comparison with the standard four-pulse HYSCORE experiment is shown in Figure S2.) The spectrum shows three series of cross-peaks (red arrows in Figure 4a) centered symmetrically around ν_Al_ = 13.59 MHz. In addition, a peak (blue arrow in Figure 4a) centered at the ^31^P Larmor frequency (ν_P_ = 21.12 MHz) is observed due to remote ^31^P lattice nuclei. The contour line shapes of the cross-peaks, all of which are located on the antidiagonal centered at the ^27^Al nuclear Larmor frequency, indicate that the spectrum can be interpreted in terms of distinct hyperfine couplings dominated by relatively large isotropic hfi parameters *a*_iso_ and small dipolar couplings, in line with the DFT computations (vide infra). We thus performed spectral simulations using DFT-derived magnetic parameters as the starting point. The best simulation of the experimental HYSCORE spectrum (Figure 4b) was obtained by summing up individual simulated HYSCORE spectra (all with the same weight) obtained considering ^21^Al with *a*_iso_ values distributed within three ranges, namely, ^27^Al(1) with *a*_iso_ = 0–2 MHz, ^27^Al(2) with *a*_iso_ = 4–9 MHz, and ^27^Al(3) with *a*_iso_ = 13–17 MHz (see also Table 1). Such a dispersion of isotropic hyperfine coupling constants, i.e., “*a*-strain,” has been observed previously in both solid-state^16,39^ and molecular systems.^40,41^ It results from structural fluctuations of the ligand environment around the paramagnetic metal center, which, in our case, can be associated with different locations of Cr^5+^ in the lattice, where it experiences slightly different coordination geometries as indicated by DFT calculations (vide infra). The isotropic hyperfine couplings can thus be explained in terms of spin density transfer to ^27^Al ions in the second coordination sphere through directly coordinated oxygens and are particularly sensitive to structural variations, the values depending markedly on the M–O–Al bond angle and distance. The dipolar coupling (*T*) was adjusted starting from the DFT-computed values, and a satisfactory simulation was obtained for maximum *T* values of 1.4 ± 0.2 MHz. Above this value, additional peaks associated with multiple quantum transitions were observed in the simulation, which were not present in the experimental spectrum. Considering that the value of *a*_0_ = 3367.76 MHz for the unit spin density in the ^27^Al 3s orbital,^42^ the corresponding spin density in the Al 3s orbital is in the range ≈ 0.06–0.5%, in good agreement with other *d*^*1*^ transition-metal ions involving M(3*d*^*1*^)–O–Al linkages.^16,39^ It is interesting to note that a similar degree of spin density transfer has been observed for the isoelectronic Ti^3+^ and V^4+^ (3d^1^) toward ^31^P and ^29^Si in microporous aluminophosphate and silicalites, and it appears to be a distinctive feature of isomorphous framework substitution.^43^ Moreover, a ridge with a maximum extension of about 2 MHz centered at the ^31^P Larmor frequency is observed. This indicates that the electron spin, localized on Cr^5+^, interacts with distant (the fourth coordination shell) phosphorus nuclei, providing firm and unambiguous evidence that chromium ions were successfully incorporated at the phosphorus sites of SAPO-5. Assuming a pure dipolar hyperfine coupling, a lower limit of the Cr–P distance of about 0.5 nm can be derived from the following equation
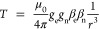
where *r* is the distance between the unpaired electron localized in the Cr d orbital and the ^31^P nucleus, in good agreement with DFT models.

**Table 1:** Spin Hamiltonian Parameters of ^27^Al and ^31^P Used for Simulating the HYSCORE Spectrum and Cluster-Computed ***g*** and Periodic ^27^Al hfi Tensor Elements at the B3LYP-D3(ABC) Level of Theory Relative to the Atomistic CrSAPO-5 Models^a^

		*g_x_*	*g_y_*	*g_z_*	Nuclei	*a*_iso_	*T_x_*	*T_y_*	*T_z_*
simulated		1.953 ± 0.003	1.972 ± 0.001	1.985 ± 0.001	^27^Al (1)	0–2	–0.5 ± 0.2	–0.5 ± 0.2	1.0 ± 0.4
					^27^Al (2)	4–9	–0.9 ± 0.3	–0.9 ± 0.3	1.8 ± 0.6
					^27^Al (3)	13–17	–1.4 ± 0.2	–1.4 ± 0.2	2.8 ± 0.4
					^31^P	≤0.1	–0.5 ± 0.2	–0.5 ± 0.2	1.0 ± 0.4
computed	Near-Si pair	1.953	1.979	1.987	^27^Al_1_	13.4	–1.6	–1.5	3.1
					^27^Al_2_	15.0	–1.4	–1.6	3.0
					^27^Al_3_	8.1	–0.9	–1.3	2.2
	Far-Si pair	1.955	1.977	1.990	^27^Al_1_	14.3	–1.5	–1.4	2.9
					^27^Al_2_	9.5	–1.4	–1.1	2.5
					^27^Al_3_	10.5	–1.1	–1.6	2.7
					^27^Al_4_	19.0	–1.9	–1.6	3.5
	Split-Si pair	1.952	1.978	1.985	^27^Al_1_	13.9	–1.7	–1.6	3.3
					^27^Al_2_	11.4	–1.2	–1.4	2.6
					^27^Al_3_	6.7	–0.8	–1.2	2.0

a Ranges of *a*_iso_ values used in the simulation are given for the simulated spin Hamiltonian parameters. The numbering of the atoms refers to the labeling shown in Figure 6. All of the hyperfine coupling values are given in MHz.

### DFT Calculations

HYSCORE spectra clearly prove the incorporation of Cr^5+^ at P^5+^ framework sites of SAPO-5. DFT modeling was then carried out to provide further insights into the electronic and geometrical structures of CrSAPO-5 and translate the experimentally obtained spectra in terms of atomistic model structures.

**Figure 5:**
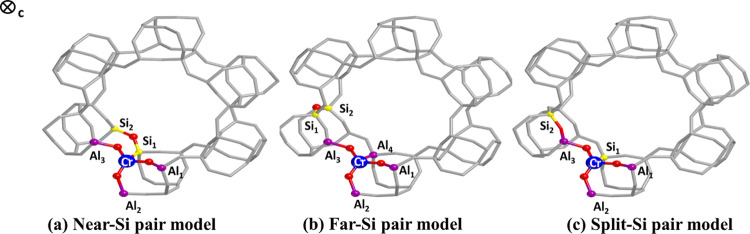
Fully optimized structures of CrSAPO-5 periodic models at the B3LYP-D3(ABC)/pob-TZVP-rev2 level of theory: in (a), the Si pair is located close to the Cr site; in (b), it is located far from the Cr; and in (c), the two Si atoms are split from each other. The *c*-axis of the unit cell points toward the page.

Three models with different Si distributions were built based on the experimental findings: they all consist of a single Cr^5+^ ion per unit cell substituting a P^5+^ framework site with two Si^4+^ replacing an Al^3+^–P^5+^ framework ion pair. Models with single silicon substitution, which involves the presence of an acidic hydroxyl group nearby to compensate for the negative charge, were neglected because no proton signals were observed in the experiments. Therefore, the substitution of aluminum and phosphorus by a silicon pair is mandatory to preserve the charge neutrality of the unit cell. Since a thorough investigation of the energetics associated with all of the possible Si pair replacements is not the object of this work, we decided to consider just three representative cases (Figure 5). In the first one, the two Si atoms are close to each other and to the Cr ion (Near-Si pair model); in the second one, the silicon ions are far from the Cr but always close together (Far-Si pair model); and in the third one, the Si pair is split to leave one silicon near the chromium and the other one far from it (Split-Si pair model).

The relative stability of the fully relaxed structures reported in Figure 5 was analyzed by taking into account their relative electronic energy per unit cell at the minimum point (Δ*E*): the lower this value, the more stable is the model and the more likely the corresponding Si distribution should be. According to the level of theory employed, the Near-Si pair model is the one with the lowest energy (see Table 2). The Far-Si pair and the Split-Si pair distributions are 19.7 and 78.2 kJ/mol higher in energy than the Near-Si pair model, respectively. The interpretation of these energy differences lies mainly in an electrostatic effect. Silicon pairs tend to stay close to each other rather than separated because of the better neutralization of the charge excess due to their framework incorporation. When they are isolated, like in the Split-Si pair model, the charge compensation is not as well balanced as when they are neighbors due to the larger distance between the silicon sites. This result is in line with the work of Nabhan et al. on SAPO-5 molecular sieves.^13^ In fact, they proved with ^29^Si magic-angle-spinning nuclear magnetic resonance (MAS NMR) that silicons in SAPO-5 materials are usually linked to neighboring Si atoms. The electrostatic effect related to the reciprocal distance of silicon atoms in CrSAPO-5 periodic models accounts for the major part of the energetic differences between Near-Si pair and Split-Si pair models. However, the increase of the electron energy when Cr is surrounded by only Al atoms can be interpreted after a careful analysis of the electronic and geometrical structures of the CrO_4_ moiety. The most relevant geometrical parameters of CrO_4_ units are enlisted in Table 2.

**Table 2:** Predicted Relative Electronic Energy Per Unit Cell, Cr–O Bond Lengths, and O–Cr–O Angles of the CrO_4_ Moiety in CrSAPO-5 Periodic Models Optimized at the B3LYP-D3(ABC)/pob-TZVP-rev2 Level of Theory^a^

models	Δ*E* (kJ/mol)	Cr–O (Å)	O–Cr–O (deg)
Near-Si pair	+0.0	1.68	108.5
		1.69	106.9
		1.66	116.5
		1.75	109.2
			106.8
			108.5
Far-Si pair	+19.7	1.69	107.2
		1.69	111.9
		1.68	109.1
		1.70	110.7
			109.7
			108.2
Split-Si pair	+78.2	1.67	107.6
		1.68	115.3
		1.65	108.3
		1.77	110.6
			105.9
			108.6

a The relative electronic energies are written with respect to the structure with the lowest energy (*E*_0_ (Near-Si pair model) = −11318.44332323 au cell^–1^).

In all of the models, chromium assumes a distorted tetrahedral geometry, whereas in the Far-Si pair model, the deviation from the tetrahedron is less pronounced, when Si is near Cr, as in Near-Si pair and in Split-Si pair models, and the Cr–O bond close to Si is longer than the others (see Table 2). This elongation in conjunction with the increase up to 115–116° of one of the O–Cr–O angles leads to a more distorted tetrahedral coordination, which is reflected in the rhombicity of the ***g***-tensor (Table 2).

Although computation results clearly show a preference for silicon distribution in the CrSAPO-5 framework, it has to be considered that the energies at stake during the synthesis procedure are relatively high. Therefore, the simultaneous presence of all three distributions considered cannot be excluded in the real catalyst as well as the presence of other structural defects inside the framework. Moreover, the synthesis process is likely to be controlled to a large extent by the kinetics of the reaction rather than purely thermodynamic factors. Nonetheless, as pointed out by Catlow,^2^ the thermodynamics of the system is expected to play a significant role in determining the final structure of the SAPO, particularly relating to the location of the Si.

### Computation and Simulation of the Spin Hamiltonian Parameters

**Figure 6:**
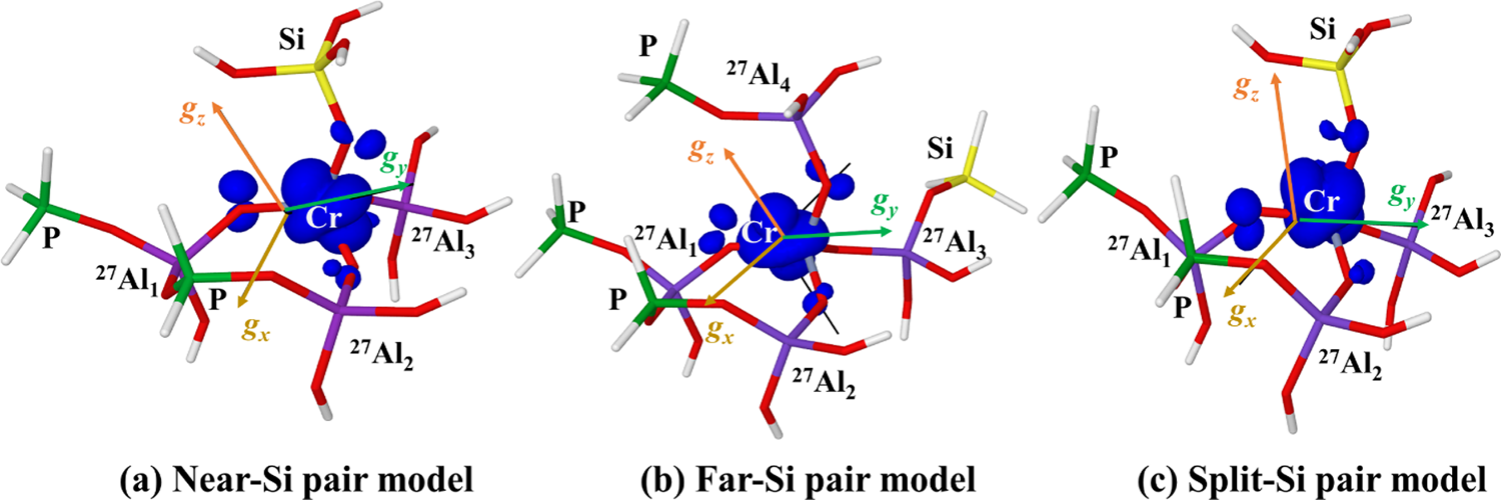
B3LYP-D3(ABC) spin density plotted on (a) Near-Si pair, (b) Far-Si pair, and (c) Split-Si pair cluster models (contour level, 0.005) together with the orientation of the computed ***g***-tensors.

**Figure 7:**
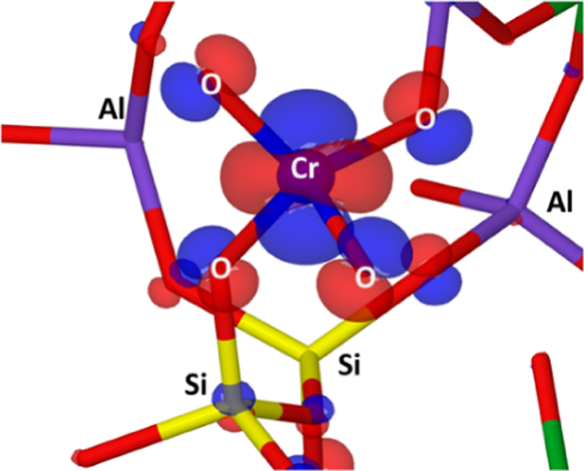
B3LYP-D3(ABC)/pob-TZVP-rev2 SOMO plotted on the Near-Si pair periodic model (contour level, 0.2).

Before discussing the simulation of HYSCORE spectra using the computed EPR parameters, let us briefly comment on the orientation of the three principal components of the calculated ***g***-tensor with respect to the distribution of the electronic spin density. This is shown in Figure 6. The majority of the spin density dwells on a molecular orbital with significant d_z_^2^ character of chromium, whereas the p orbitals of the oxygen ligands contribute to the remaining part, in agreement with the singly occupied molecular orbital (SOMO) structure. As an example, the computed singly occupied molecular orbital (SOMO) is illustrated in Figure 7 for the Near-Si pair periodic model. The orientation of the ***g***-tensor is affected by the localization of the unpaired electron: its prevalent presence on the d_z_^2^ orbital means that the *g*_*z*_ component points directly outside the orbital lobe.

A comparison of the cluster-computed principal values of the ***g***-tensors with the simulated ones is reported in Table 1, and the values are in line with the experimental results.

For an axially distorted tetrahedral symmetry, assuming pure atomic *d* orbitals within crystal field (CF) theory, the spin Hamiltonian parameters for a d_z_^2^ ground state are given as
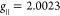

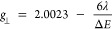
where λ is the spin–orbit coupling parameter and Δ*E* is the energy separation of the state above the ground state, leading to *g*_||_ (i.e., *g*_*z*_) > *g*_⊥_ (i.e., *g*_*x*_, g_*y*_). This simple picture, however, cannot be reconciled with the experimental spectrum, which shows significant rhombicity and large departure of *g*_*z*_ from the free-electron value (2.0023). This indicates that the simple CF model is inadequate for CrO_4_^3–^ and covalency must be considered, as pointed out also from the significant spin density delocalization over the oxygen predicted by DFT (Figure 7). Analysis of the DFT-computed ***g***-tensor (Figure 6) indicates that the largest component (*g*_*z*_) is oriented approximately along the lobe of the d_z_^2^ orbital; however, the low symmetry of the sites (Table 1) implies an appreciable admixture of the d_x^2^-y^2^_state into the d_z_^2^ ground state. Similar, rather unusual, ***g***-tensor principal values characterized by significant rhombicity of the tensor have been reported from single-crystal studies of CrO_4_^3–^ featuring the d_z_^2^ ground state in low-symmetry hosts.^44^ As an example, in the case of CrO_4_^3–^ species in YPO_4_ and YVO_4_ featuring the *C*_2*v*_ symmetry, as reported by ref (36), a small amount of d_xy_ is mixed in d_z_^2^, allowing direct spin–orbit coupling with d_x^2^-y^2^_, which, in turn, explains the large deviation of *g*_*z*_ from *g*_e_ (2.0023) and the rhombicity of the tensor.^45,46^ In our case, the larger distortion of the tetrahedral cluster is observed when Si is located close to the Cr^5+^ ion (Near- and Split-Si pair models in Figure 5); consequently, these models show the largest departure of *g*_*z*_ from *g*_*e*_ and the highest rhombicity of the ***g***-tensor.

The three sets of computed ***g***-tensor components for the corresponding cluster models qualitatively agree with the ESE–EPR spectrum shown in Figure S1b; however, the broad peak makes it difficult to experimentally assign a specific structure. For this reason, we can merely conclude that the different Si distributions modeled produce ***g***-tensors that fall inside the range of the experimental measurement.

The computed ^27^Al hfi tensor elements collected in Table 1 are mainly dominated by the isotropic (*a*_iso_) term, in good agreement with the experimental findings (simulated values in Table 1). The *a*_iso_ value is found to change significantly for the different Al atoms around chromium, reflecting the different degrees of spin transfer, which, in turn, is determined by both the bond angle and distance. The dipolar contribution is far less sensitive, leading to similar values for all ^27^Al nuclei regardless of which model is considered.

Considering the different models, *a*_iso_ for the aluminum atoms (^27^Al_1_, ^27^Al_2_, ^27^Al_3_, and ^27^Al_4_) can be gathered in three groups: ^27^Al_1_ is characterized by an isotropic hfi constant with values ranging from 13.4 up to 14.3 MHz; ^27^Al_2_ has values around 9.5 and 15 MHz; and ^27^Al_3_ has the lowest values, ranging from 6.7 to 10.5 MHz. The Far-Si pair model also contains another Al close to Cr, labeled ^27^Al_4_, with the highest isotropic coupling of 19 MHz. Although an unambiguous assignment of the DFT-computed ^27^Al hfi to the experimental ones is not straightforward, the range of the observed hyperfine couplings covers the calculated values very well, endorsing the efficacy of the models in describing the real system. This is reflected by the simulation of the ^27^Al HYSCORE spectrum (shown in Figure S3) obtained using the computed hyperfine coupling constants reported in Table 1. The good agreement with the experimental findings strongly validates the computational models. The DFT-computed ^27^Al hfi constants explain all of the cross-peaks in the experimental spectrum apart from the ones related to aluminum with *a*_iso_ in the range 0–2 MHz. However, *a*_iso_ values close to 0 are clearly assigned to remote (matrix) nuclei, couplings of the order of 2 MHz imply some spin density on the Al, which may be associated with the presence of structural defects inside the framework.

## Conclusions

The main findings obtained from this study of Cr^5+^-doped SAPO-5 materials can be summarized as follows.

(a) X-band CW-EPR spectra of as-synthesized CrSAPO-5 show the characteristic spectrum of Cr^3+^ species. After calcination, the presence of Cr^5+^ species is demonstrated by the presence of a new EPR signal resonating at *g*_*z*_ = 1.985 ± 0.001, *g*_*y*_ = 1.972 ± 0.001, and *g*_*x*_ = 1.953 ± 0.003.
(b) HYSCORE spectra of Cr^5+^ species reveal the presence of distinct ^27^Al cross-peaks, reported here for the first time, associated with relatively large hfi dominated by the Fermi contact term, implying spin density transfers ranging in the interval 0.2–0.5%, consistent with Cr–O–Al linkages. The spectra also show the presence of a ^31^P signal due to the hyperfine coupling with distant (>0.5 nm) ^31^P nuclei. The presence of large hyperfine couplings to ^27^Al and small coupling to ^31^P provides compelling evidence for framework substitution of Cr^5+^ at phosphorous sites.
(c) Three different possible models have been considered to account for the experimental results, consisting of Cr^5+^ species isomorphously substituted at P^5+^ sites with different Si distributions. DFT-computed EPR data for the three models reproduce well the experimentally observed ***g***-tensor and ^27^Al hfi parameters, indicating that the experimental results can be explained considering different isotopomers featuring different Si localizations. This is in agreement with the distribution of *a*_iso_ parameters (*a*-strain) deduced from the simulation of the HYSCORE spectrum.

## Supporting Information Available

Experimental and simulated X-band CW-EPR and Q-band ESE-detected EPR spectra of the calcined CrSAPO-5; Q-band four-pulse HYSCORE spectrum of the calcined CrSAPO-5; periodic-computed ^27^Al hfi; cluster ***g***-tensor at the PBE0-D3(ABC) level of theory; and atomic coordinates of cluster and periodic models (file type DOCX) (PDF)

## Abbreviations

EPR: electron paramagnetic resonance
HYSCORE: hyperfine sublevel correlation spectroscopy
DFT: density functional theory
SAPO: silico-aluminophosphate
ESEEM: electron spin echo envelope modulation
