# Supplementary material for: Chromium Environment within Cr-Doped Silico-Aluminophosphate Molecular Sieves from Spin Density Studies
Source: J Phys Chem C Nanomater Interfaces. 2021 Apr 7;125(15):8116–24. doi: 10.1021/acs.jpcc.0c09484 (PMC8162410; doi:10.1021/acs.jpcc.0c09484)
Supplement: Supplementary file 1 — jp0c09484_si_001.pdf [file jp0c09484_si_001.pdf]

Chromium Environment within Cr-doped Silico-Aluminophosphate Molecular Sieves from Spin Density Studies

Yu-Kai Liao,[1,2]‡ Paolo Cleto Bruzzese,[1,2]‡ Martin Hartmann,[3] Andreas Pöppl[2] and Mario Chiesa[1]**

[1]Dipartimento di Chimica, Università di Torino and NIS Centre, via P. Giuria 7, 10125, Torino, Italy

[2]Felix Bloch Institute for Solid State Physics, Universität Leipzig, Linnéstr. 5, 04103 Leipzig, Germany

[3] Erlangen Center for Interface Research and Catalysis (ECRC), Egerlandstr. 3, 91058 Erlangen, Germany

Corresponding authors: poeppl@physik.uni-leipzig.de, mario.chiesa@unito.it

Supporting Information

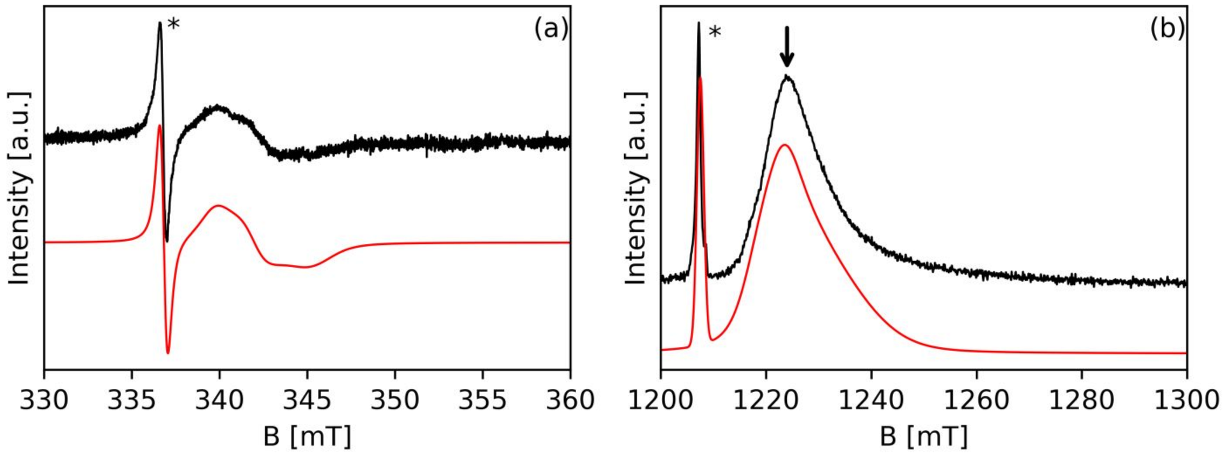

Supporting Figure S1. The experimental (black) and simulated (red) of (a) X-band cw and (b) Q-band ESE EPR spectra of the calcined CrSAPO-5 at *g*~2 region. The arrow marks the field position of the HYSCORE measurements and the asterisks mark the radical impurity.

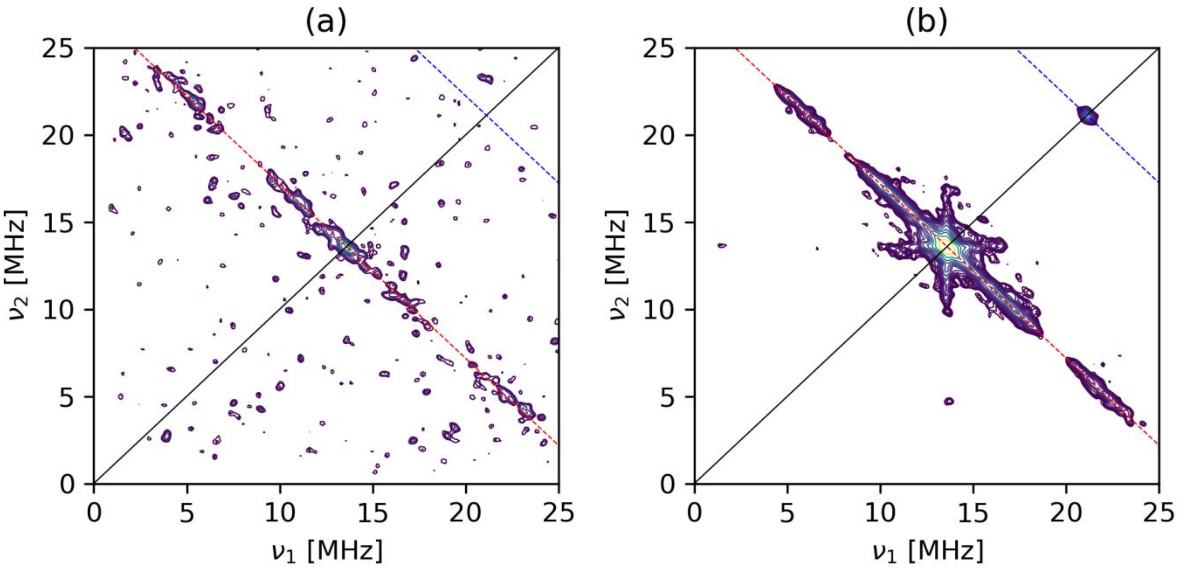

Supporting Figure S2. Comparison of Q-band (33.8 GHz) (a) standard 4-pulse HYSCORE spectrum superimposed with interpulse delays (τ = 110, 116, 146 ns) and (b) 6-pulse HYSCORE spectrum superimposed with interpulse delays ((τ = 110, 146 ns). Both spectrum measured at T = 40 K and field position as marked in Figure S1b. The red dashed lines mark the Larmor frequency of ^{27}Al (ν_{Al} = 13.59 MHz) and the blue dashed lines mark the Larmor frequency of ^{31}P (ν_P = 21.12 MHz).

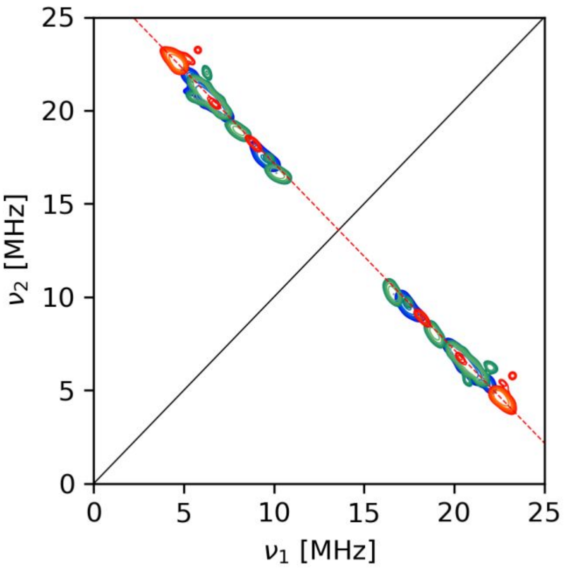

Supporting Figure S3. Computer simulation obtained using the DFT computed ^{27}Al *hfi* tensors. The different colours refer to the Al species of the different DFT models (Near-Si pair in blue, Far-Si pair in red, and Split-Si pair in green).

Supporting Table S1. Cluster computed *g* and periodic ^{27}Al *hfi* tensors elements at PBE0-D3(ABC) level of theory relative to the structures in Figure 4 (***A***-tensors) and Figure 5 (*g*-values). All the hyperfine coupling values are given in MHz. The numbering of the atoms refers to the labelling shown in Figure 4 and 5.

Models	g_x	g_y	g_z		a_{iso}	T_x	T_y	T_z
Near-Si pair	1.949	1.977	1.985	$^{27}Al_1$	+13.9	-1.9	-1.4	+3.3
				$^{27}Al_2$	+15.7	-1.6	-1.3	+2.9
				$^{27}Al_3$	+7.2	-1.2	-0.7	+1.9
Far-Si pair	1.952	1.976	1.988	$^{27}Al_1$	+15.1	-1.7	-1.3	+3.0
				$^{27}Al_2$	+6.9	-1.3	-0.8	+2.1

				$^{27}Al_3$	+12.2	-1.5	-1.1	+2.6
				$^{27}Al_4$	+25.2	-2.0	-1.7	+3.7
Split-Si pair	1.948	1.977	1.983	$^{27}Al_1$	+14.5	-1.9	-1.5	+3.4
				$^{27}Al_2$	+13.1	-1.5	-1.1	+2.6
				$^{27}Al_3$	+5.4	-1.1	-0.6	+1.7

Atomic coordinates of the optimized CrSAPO-5 models

Close-Si pair model

Periodic

Cell:

A	B	C	ALPHA	BETA	GAMMA
13.6516	13.7144	8.2855	90.25	89.79	120.03

Coordinates:

Al	6.556879263741	0.941554463270	-4.163114007602
P	6.385112219649	0.722949792081	1.038452027769
P	-4.056813483465	-2.314439617443	1.074723663105
Si	-2.666650489169	3.056937782326	1.090512789312
P	-0.050860430073	-6.078283112738	1.108957695062
P	2.548993596032	4.601190386851	1.064506648491
P	5.173913627835	-4.547380200321	1.073022043207
Cr	-0.381822480161	4.934616865342	-2.974190122153
P	-4.964590162314	4.933513939404	-3.109737083889

P	-2.692910036522	-4.689124470356	-3.132951384092
P	5.191498694123	3.529092881204	-3.064305103037
P	-4.322205554734	0.700548301995	-3.066332895613
P	2.853016955772	-6.064552810801	-3.099076045066
Al	-4.002033260062	-2.118594490129	-4.122992157724
Al	-2.714478868143	3.105855552555	-4.072854663435
Al	-0.051692972581	-5.841533658310	-4.102012938066
Al	2.615866565264	4.798264677490	-4.148902729959
Al	-6.569147004253	2.470072797724	-4.183653134157
Si	-0.325993362632	4.801887244661	0.171410819662
Al	6.792022007874	-2.118301463207	0.110110690116
Al	-2.795855129044	-4.953867079561	-0.029986084402
Al	5.152152976948	3.344610620479	0.053138161926
Al	-4.265283377280	0.628881409652	0.012467619924
Al	2.851940301046	-6.235462047502	0.007677262565
O	5.939825814796	-0.737043249691	0.802096200275
O	-3.682165021768	-0.869379249089	0.698513659926
O	-1.076416130798	3.513922093076	0.744627307536
O	-1.217145011087	-5.179135114763	0.678362599435
O	3.539885306323	3.466205822934	0.741698810995
O	3.691710661028	-4.924569415459	0.753316752029
O	-1.099310064826	3.504597333608	-3.400375167636
O	6.212064204425	-0.670167976515	-3.662793562789

O	-1.240280239636	-4.663334196068	-3.693559066596
O	3.785123190112	3.605678477567	-3.723505177759
O	-3.585668140848	-0.481808561634	-3.764653351477
O	3.597818361713	-4.815158093514	-3.621288303462
O	6.018523678380	1.185153275370	2.479561870403
O	-3.624551475490	-2.610613037003	2.540603577429
O	-2.651188027568	2.522808759213	2.625698609286
O	0.442751316433	-5.711138290595	2.528790431922
O	1.997116497105	4.483123188130	2.509403978988
O	5.242241382377	-3.967370381751	2.514943245862
O	-0.821229301221	5.304649863893	-1.315319106571
O	-4.622665360921	5.197359619073	-1.624683937464
O	-2.698210983293	-5.338253290855	-1.721678581906
O	5.092483352184	3.951391385415	-1.573067873420
O	-4.759724490579	0.284021734958	-1.638386857486
O	3.301339206760	-6.431252737311	-1.656932491514
O	5.662594265446	1.679666716989	0.039137241393
O	-3.305538676284	-3.303315408609	0.136417497020
O	-3.097779728292	1.868428791217	0.073351348847
O	1.112383112522	-5.982485077303	0.095641667916
O	1.312467408992	4.468709988601	0.080905398654
O	5.650915063097	-3.461326563966	0.071902445942
O	1.293482236356	4.721373187309	-3.000951625035

O	-6.012768654254	3.789515106604	-3.209818350521
O	-3.199012120287	-3.224667308451	-3.035953555447
O	5.703501082742	2.067458462298	-3.143808660249
O	-3.360725609011	1.915208144620	-2.982474743276
O	1.328275664325	-5.739779731999	-3.067104662491
O	-3.912589556206	-6.015644374969	0.767882325378
O	-5.599891147483	-2.531150673253	0.940588871058
O	-3.581502971675	4.395671789576	0.904541607108
O	-0.502737279550	6.126666641050	1.173515441858
O	3.220024780074	5.979984871949	0.840680431657
O	-5.746509178528	1.044973834571	0.879151595231
O	-0.721729195179	6.261013878802	-3.944162268031
O	-3.662515061868	4.568105178631	-3.883441659342
O	-3.578807366008	-5.536210467870	-4.091245668390
O	-5.720315906937	-2.328472646870	-3.891638005683
O	-5.580293904891	1.061370324303	-3.913685735551
O	3.194370786704	6.444079113220	-4.065136214951

Cluster

H	3.8268438575	-3.2176218654	-1.5063577803
H	0.4279448641	-3.2522736999	3.0629886554
Cr	0.8099826083	0.1540555193	-0.1671540715
H	4.9022995564	0.4148363691	0.1878404426

H	2.0271378323	0.5098213396	4.1081048419
Al	3.4975781912	-0.7243340533	-1.5806529755
Al	0.2412716797	-0.7137469850	2.8351087399
O	1.7600640314	-0.2966392080	-1.4460588830
O	1.1930974929	0.0159006574	4.0717858103
O	3.7153508610	-2.3564416303	-1.9386052955
O	0.9351882335	1.8936452544	0.0328632454
O	1.1264408762	-0.5944853520	1.3009236897
O	4.2119678164	-0.2192769204	-0.0616438755
H	4.8043986673	0.2926612819	-3.5026748857
O	4.1112946652	0.3243025792	-2.8248064099
H	1.9867868324	4.0814165239	-2.1549580771
H	-0.2781931305	4.3653280199	1.3707864387
Si	0.2819176368	3.2112888006	-0.7056811151
O	1.0905788511	3.7605004776	-1.9686139187
O	0.1314451073	4.3665119349	0.4915281647
H	-1.2747399177	-3.9825888191	-0.3365754345
P	-4.4582696006	-1.0329031395	-2.1374776377
P	-2.7324494794	-0.2487404291	2.1765413419
Al	1.9264594701	-1.5203267971	-0.4721291620
O	-2.9874605505	-1.4314978147	-1.8274048175
H	-3.5386752671	-0.7512216982	3.3955304631
O	-0.0429741043	-2.4080556156	3.1431831144

O	-1.0681130241	-3.0470027611	-0.4878502009
H	-4.6470191262	0.4903632782	-1.9009156474
H	-3.3500134787	1.0479506150	1.5828690857
O	-0.8001234408	-0.1797264880	-0.5531372346
H	-4.7626971627	-1.3562046194	-3.6232913728
O	-1.2847556816	0.0766748838	2.6557315523
H	-5.3782941435	-1.8300246123	-1.1749701600
O	-2.7143080113	-1.3580318885	1.0781431176
H	-2.1120922139	3.2780689858	-1.2756542793
O	-1.2669996383	2.8083201407	-1.1978432187

Far-Si pair model

Periodic

Cell:

A	B	C	ALPHA	BETA	GAMMA
13.6927	13.6241	8.2410	90.37	89.29	119.67

Coordinates:

Al	6.583071046310	1.042956896163	-4.119686164741
P	6.547713046885	0.679255882246	1.033632921297
P	-4.060586031100	-2.344178906681	1.117881821144
P	-2.704918546408	2.908941108570	1.121231527827

P	-0.021241657763	-6.105018562366	1.126250012189
P	2.601292626206	4.387140358328	0.993903186763
P	5.251489890602	-4.590919086518	1.129044275978
Cr	-0.442078043892	4.952174849914	-3.012988830049
P	-5.052116577873	4.964250889402	-3.187244156435
P	-2.749784505004	-4.584540979828	-3.118767845786
P	5.218852728072	3.650093338506	-3.017726047870
Si	-4.366219732504	0.814911593563	-3.000258327711
P	2.767444126726	-5.911857384627	-3.120791151013
Al	-4.030263673088	-1.980636253264	-4.031992746350
Al	-2.807040321769	3.181022034236	-4.039902812562
Al	-0.125940712775	-5.705336802545	-4.058279794597
Al	2.587237627478	4.878194620177	-4.167045767503
Al	-6.689302518790	2.487111426620	-4.148013964933
Al	-0.327056365708	4.619275440097	0.166223304436
Al	6.837022934300	-2.191958142177	0.043617359732
Al	-2.719464337207	-4.937423623071	-0.033467962954
Al	5.247724165109	3.263998771512	0.072845780036
Si	-4.312200586486	0.543414433385	0.074521408832
Al	2.883066072668	-6.345096445477	-0.037393281190
O	6.119355435679	-0.798045499042	0.811857961138
O	-3.705201036111	-0.845107175608	0.731353901631
O	-1.233753619466	3.266989341334	0.813845576462

O	-1.134790511332	-5.156846900643	0.617914265818
O	3.649466461190	3.259170955920	0.776085581872
O	3.866373789437	-5.138815548617	0.750315871550
O	-1.131130323388	3.584627517788	-3.717288769513
O	6.210414082862	-0.519213632398	-3.505382112447
O	-1.310373017881	-4.489665454386	-3.701545476931
O	3.788900059820	3.785065711965	-3.592576417485
O	-3.534863961213	-0.403617528545	-3.678490065437
O	3.507347482546	-4.638089437531	-3.594876513564
O	6.116614021363	1.147949096612	2.449266807150
O	-3.626510683547	-2.548086645794	2.579348728015
O	-2.890666994530	2.454866153999	2.581404030130
O	0.434849936442	-5.648121181854	2.537801596846
O	2.104287193153	4.389319272836	2.466336661931
O	5.249370497454	-3.943231149402	2.523245812107
O	-0.973504199416	5.045530029370	-1.400759059159
O	-4.582277477429	5.076678249363	-1.706360607180
O	-2.724204521826	-5.287770380664	-1.731878321385
O	5.212859835135	3.989062490637	-1.499697830843
O	-4.794752008935	0.345218021523	-1.436037349339
O	3.275903389369	-6.373253322651	-1.723775432655
O	5.862295389757	1.623800598969	-0.003224092666
O	-3.246695216332	-3.274677860128	0.191085623293

O	-3.095306342905	1.666560827526	0.208934723110
O	1.186435800085	-6.010456628526	0.152830808008
O	1.361679603631	4.163105556332	0.079120468855
O	5.745031489766	-3.573096834933	0.064962495446
O	1.239844359131	4.805698859927	-3.051870557716
O	-6.172926739489	3.899235506232	-3.277327109693
O	-3.311457858973	-3.149367764197	-2.945553935627
O	5.675724820796	2.177311907228	-3.182350436894
O	-3.432376023222	2.140580684152	-2.846656576601
O	1.251086429440	-5.571210538422	-3.011714283883
O	-3.826190149618	-6.019915160682	0.771879416135
O	-5.587275700608	-2.549175009072	0.936391784369
O	-3.616307022787	4.113007605119	0.781504128328
O	-0.482903353182	6.047389528876	1.168319135854
O	3.302965517555	5.741503140869	0.660586877231
O	-5.587215528402	1.010418453686	1.033885285098
O	-0.838638172011	6.359822053071	-3.854459292143
O	-3.822540717158	4.623558788859	-4.084579393430
O	-3.634648534651	-5.412644588628	-4.097446477262
O	-5.769633393346	-2.171555513071	-3.886510439559
O	-5.744409963109	1.115377802174	-3.807563009104
O	3.068427090495	6.561152840979	-4.182772216884

Cluster

H -1.7802011924 -4.0190374611 0.7685268223

H -2.2951178449 -2.6584067058 -2.9276659110

P -4.2333235289 1.5837688990 -3.3831396398

P -3.9010385908 -0.2359631282 0.9553903976

Al -2.4944212530 -0.3999456081 -1.7112205785

O -3.0495175280 0.7080420015 -2.9083960572

H -5.1563569759 -0.8970554532 1.5733963390

O -2.1076662060 -3.2203225899 1.2109307322

O -1.9618217362 -1.8666030066 -2.4772654159

H -4.2845772846 2.8986361140 -2.5537712895

H -4.0654236269 1.3090750148 0.8604517596

O -1.1888055152 0.3795133252 -0.8438623328

H -3.9998422157 1.9587190106 -4.8697226778

O -2.6891232147 -0.5521539763 1.8819741386

H -5.5494357163 0.7880577306 -3.1977071001

O -3.6718278816 -0.8077545259 -0.4817308865

Si 5.5965479160 0.4250068922 -1.9903056790

H 5.3513082347 0.3245093782 -3.5921292465

H 6.2839288880 1.9320495607 -1.6626594365

H 6.6464748575 -0.7049339197 -1.4781360343

H 3.0820160851 -3.1546959471 -2.2315366963

Cr 0.2529034506 0.0201087425 -0.0413331194

H	3.9803068016	-1.2925177701	1.3156865399
H	-0.0682357636	-1.8813483030	3.9986336127
Al	3.0897784965	-0.9128094227	-0.9719309628
Al	-1.4622094620	-1.7772107297	1.9403133748
O	1.4318690086	-0.3793551970	-1.1777351289
O	-0.9193457948	-2.0496906477	3.5648449893
O	3.3698308468	-2.2376514073	-2.1008004155
O	0.7858437571	1.3848173498	0.8210615742
O	4.1834211138	0.3745822610	-1.1818283597
O	-0.0677256933	-1.2613927709	1.0082907535
O	3.3560040082	-1.5902401491	0.6356075685
H	2.8802230009	3.9707190328	0.4483281141
Al	0.4928241838	3.1069012590	0.7618255873
O	1.9426989507	3.9181476289	0.2050486396
O	0.0609849924	3.5892959816	2.3893019877
H	-0.6353003025	3.3459943507	3.0192949278
P	-1.9725881142	4.5606844692	-0.2979049488
H	-2.4237015815	4.9083561726	-1.7450172740
H	-1.4386045562	5.8292566345	0.4236950017
O	-0.8207801592	3.5135900490	-0.3228898787
H	-3.2207353204	3.9991565718	0.4535282073

Split-Si pair model

Periodic

Cell:

A	B	C	ALPHA	BETA	GAMMA
13.6349	13.7560	8.2639	90.39	89.29	120.39

Coordinates:

Al	6.490494833782	1.019937770939	-4.139937952183
P	6.411406979220	0.624599508267	1.023239785226
P	-3.994998072545	-2.395396829981	1.142427515697
P	-2.637167865388	2.997850059243	1.068534654239
P	-0.000760914617	-6.211475281430	1.136959502051
P	2.569062531353	4.485262870107	1.022947661341
P	5.198263658864	-4.617932126006	1.150628138275
Cr	-0.462376803974	5.055835429567	-2.992926751245
P	-4.998897630679	5.027001747541	-3.180971074862
P	-2.710436883919	-4.598847829588	-3.133622421872
P	5.124117239399	3.633632359678	-3.030852938414
Si	-4.332669444031	0.787261452209	-3.037263575526
P	2.783976101425	-5.979023650211	-3.116075199849
Al	-3.978734482795	-1.996459953830	-4.063983453300
Al	-2.797317570567	3.211218534555	-4.055669839483
Al	-0.107062718465	-5.744627071569	-4.054454392820

Al	2.565377609948	4.952994182531	-4.173327463685
Al	-6.599864178617	2.555160336528	-4.193038691921
Si	-0.315518921997	4.743058156302	0.168975325666
Al	6.758218928929	-2.233951511532	0.041846134851
Al	-2.719338959061	-5.004762975932	-0.048652463926
Al	5.162020818494	3.243617398014	0.076836596891
Al	-4.239548259430	0.530420703419	0.056200360397
Al	2.921030819874	-6.380940730251	-0.033082386178
O	5.993956573067	-0.851275397659	0.794555215602
O	-3.636055370309	-0.938350958873	0.782910161201
O	-1.122848147015	3.438750443685	0.750205206039
O	-1.147211308887	-5.334655973737	0.632128150793
O	3.529513358199	3.307750688036	0.769212797421
O	3.770845710922	-5.119590112810	0.769936537966
O	-1.126176872870	3.657781520602	-3.565735518024
O	6.158003620157	-0.539779622794	-3.493662764360
O	-1.270366110756	-4.517104270793	-3.735677592933
O	3.696842630183	3.784301211265	-3.633147353733
O	-3.502405400013	-0.401502920558	-3.803021161669
O	3.483139412057	-4.689764928703	-3.584001560073
O	6.016977339485	1.078170268677	2.453299434487
O	-3.608972443402	-2.695592976271	2.613405414349
O	-2.654634232562	2.630480953331	2.560644498252

O	0.446582960635	-5.797201255526	2.553214815091
O	2.024032450529	4.503126203432	2.472947190217
O	5.140429017199	-3.902883663633	2.524817593355
O	-0.882495975243	5.221258385584	-1.279839973516
O	-4.510842501546	5.136367339654	-1.704782189202
O	-2.644906245349	-5.319900586239	-1.753551867765
O	5.064982591322	3.930591789474	-1.507442253857
O	-4.765859134986	0.307891693054	-1.533163002097
O	3.277120552669	-6.457650675874	-1.721729529760
O	5.692110359288	1.574572115666	0.012975334158
O	-3.196505782508	-3.365490182829	0.218954117469
O	-2.958878306357	1.773463230365	0.202707171031
O	1.180640701190	-6.177138714701	0.148362433648
O	1.282961919544	4.296395924117	0.088411312097
O	5.665204712109	-3.587930443363	0.071651632983
O	1.213759610471	4.906898236261	-3.045314053324
O	-6.114710144596	3.962875362965	-3.279456109983
O	-3.265146227586	-3.168913776564	-2.949440215356
O	5.569965560892	2.161079023193	-3.221791928291
O	-3.363804046244	2.116427891983	-2.914599418822
O	1.255226214401	-5.667534638368	-3.002803714839
O	-3.815334965540	-6.116950912839	0.720405595192
O	-5.529655782338	-2.649557512937	0.949255059522

O	-3.521862854660	4.235297096159	0.766273933995
O	-0.480587436438	6.020885737190	1.220263248838
O	3.270286932491	5.813119285779	0.657795640172
O	-5.565975807329	1.102540576477	1.065310515651
O	-0.873171970084	6.439194702635	-3.835028988988
O	-3.766188789559	4.680038646254	-4.081531722516
O	-3.582774739318	-5.448836365110	-4.103566834029
O	-5.719016816417	-2.206352059389	-3.860368928921
O	-5.656756292137	1.179981754562	-3.919575760105
O	3.051306468669	6.626265763952	-4.185759333122

Cluster

H	3.2774987922	-2.4122665221	-3.1732000923
Cr	0.6776677457	0.1316872495	-0.0902575693
H	4.7798717279	-0.0225502597	-0.1185946869
H	2.1923908566	-1.5237481968	3.7835314134
Al	3.1816243042	-0.0765107192	-2.0062006361
Al	0.2631628554	-1.9224896029	2.2953526998
O	1.4540285658	0.1135513382	-1.5463585598
O	1.3353586865	-1.9551517908	3.6410996808
O	3.2125691982	-1.4492367866	-3.0769822340
O	0.9308648148	1.7134961009	0.6662496538
O	3.6901366994	1.4045259921	-2.6140333494

O	1.1007623091	-1.0966850192	0.9602967154
O	4.0810785273	-0.5279678211	-0.5626004881
H	4.2990502591	1.6414017855	-3.3309811406
H	1.9671896108	4.5728067518	-0.4351349935
Si	0.2556308406	3.1944346089	0.6570610194
O	1.0646084845	4.2379506432	-0.3163128701
O	0.2653341532	3.7612314166	2.2203730299
H	0.2356459795	-4.2391016155	1.1672108976
H	-1.7741511900	-3.3696291761	-2.0278146461
P	-4.8114056635	0.1789372706	-1.8511823782
P	-2.6881498079	-1.1683451253	2.1744296999
Al	-2.2211847616	-1.1568781058	-0.8026285540
O	-3.3432036898	-0.3356620689	-1.8048903263
H	-3.3992439795	-2.0927417162	3.1793219800
O	-0.1378852263	-3.5109741531	1.6879842043
O	-1.5184982011	-2.4970138652	-1.6900513142
H	-4.9058633136	1.5126111715	-1.0607055647
H	-3.2849213721	0.2668699851	2.1521492028
O	-0.9780895546	0.0135520446	-0.3710988566
H	-5.1705212361	0.4653993146	-3.3319346377
O	-1.1919729630	-1.0584621692	2.6175015152
H	-5.7170834014	-0.9003395064	-1.2240872672
O	-2.7794016918	-1.7683140259	0.7305573986

H	-0.0738996165	3.3628546051	3.0371449476
H	-2.1390640026	3.5932597376	0.3312983657
O	-1.3067737623	3.1559447020	0.0926594845
